# Dietary eating patterns, dairy consumption, and anxiety: A systematic literature review

**DOI:** 10.1371/journal.pone.0295975

**Published:** 2023-12-28

**Authors:** Naimisha Movva, Heidi Reichert, Naushin Hooda, Lauren C. Bylsma, Meghan Mitchell, Sarah S. Cohen

**Affiliations:** EpidStrategies, A Division of ToxStrategies, LLC, Katy, Texas, United States of America; University of Petra (UOP), JORDAN

## Abstract

**Background:**

Nutrition affects both physical and mental health but evidence is mixed regarding potential associations between anxiety and diet, particularly dairy consumption. We conducted a systematic literature review (SLR) of dairy consumption and/or various dietary patterns and risk of anxiety.

**Methods:**

Literature searches were conducted in PubMed and Embase. All study designs except case reports, small case series, and SLRs were considered for inclusion. Reference lists of previously published SLRs were reviewed for any relevant additional studies. Studies of populations without dairy sensitivities exploring the association between dietary patterns and/or dairy consumption and anxiety published through May 2022 were identified using predefined eligibility criteria. Study quality was determined using the Academy of Nutrition and Dietetics Quality Criteria Checklist.

**Results:**

For this SLR, 132 studies were included; 80 were cross-sectional. Studies examined different dietary patterns (e.g., Mediterranean, gluten-free) and anxiety using various anxiety scales, with 19 studies specifically reporting on whole dairy consumption and anxiety. Dairy consumption was significantly associated with a lower risk of anxiety in 7 studies, while the remaining 12 studies showed no significant associations. Evidence was mixed for the association between various dietary patterns and anxiety, but more studies observed a lower risk of anxiety with greater adherence to “healthy” diets (e.g., Mediterranean, diet quality score, vegetarian/vegan) than a higher risk. Notable heterogeneity in study populations, time periods, geographical locations, dietary assessment methods, and anxiety scales was observed.

**Conclusions:**

The results of this SLR suggest a potential link between diet including diary consumption and anxiety, but future studies, especially with longitudinal designs that measure diet and anxiety at several timepoints and comprehensively adjust for confounders, are needed to fully understand the relationship between diet and anxiety.

## Introduction

Anxiety disorders affect up to one-third of the population based on large population-based survey across the United States (US) and Europe [1]. Nutrition is one of many factors that may affect an individual’s mental health. In a recently published scoping review of diet and anxiety, greater consumption of certain healthier foods such as fruits and vegetables, omega-3 fatty acids, and “healthy” dietary patterns resulted in less anxiety, while other factors (such as a high-fat diet and high intake of sugars) were associated with more anxiety [2]. Dairy consumption was not specifically addressed in this scoping review, but dairy consumption is an integral part of a healthy dietary pattern which represents everything individuals habitually eat and drink and the ways in which these elements act together to impact health [3]. For adults, the 2020–2025 US Department of Agriculture Dietary Guidelines for Americans recommend an equivalency of 3 cups of dairy per day [3].

A systematic review of dairy consumption and depression conducted through December 2018 reported mixed findings that depended on the gender, population, or dairy type across the 13 included studies; the authors further reported that no studies specifically related to dairy consumption and anxiety, as a secondary outcome, were identified [4]. However, observational studies describing the association between dairy consumption and anxiety have recently been identified and report inconsistent findings [5–7].

Given the high prevalence of anxiety and the potential for diet to be a modifiable risk factor for this important aspect of mental health, the purpose of this systematic literature review (SLR) is to summarize the published literature on associations between dietary patterns or eating habits and anxiety. This review also identified any publications specifically related to the consumption of whole dairy products and the risk of anxiety.

## Methods

A study protocol was developed and registered on PROSPERO (CRD42022333785) (www.crd.york.ac.uk/PROSPERO) prior to the start of the SLR. The Preferred Reporting Items for Systematic Reviews and Meta-analyses (PRISMA) [8] guidelines were followed in all aspects of the preparation, conduct, and reporting of this SLR.

### Eligibility criteria

Pre-defined study population, intervention, comparator, outcomes, and study design (PICOS) criteria were utilized to identify relevant studies. Studies of persons with a dairy sensitivity were excluded; no additional specifications on population were applied. Any studies examining a dietary pattern (no restrictions on type of pattern were made) or consumption of dairy products (milk [both regular and fermented], yogurt, or cheese) were considered for inclusion. Comparators were low or no adherence to the dietary pattern or low/no dairy consumption. Studies reporting outcomes related to risk of anxiety diagnosed using a validated scale or diagnosis code were included while studies of self-reported anxiety were excluded. Studies examining risk of anxiety in relation to adherence to a specific diet were also included. Observational studies (cohort, case-control, and cross-sectional) and randomized controlled trials (RCTs) were included. Studies not published in English, case series with <20 participants, and studies not meeting the PICOS criteria were excluded. Reference lists of relevant reviews were checked to identify any additional studies meeting the PICOS criteria that were not captured by the literature searches.

### Study identification, screening, and abstraction

Comprehensive literature searches were conducted in the PubMed and Embase databases to capture literature published through May 19, 2022. The search strategy is described in S1 Table. Study de-duplication, article screening, and data abstraction were conducted using DistillerSR software [9], which allows for the SLR management process to be transparent and auditable. Using the PICOS criteria, one reviewer screened the titles and abstracts of the identified studies. If an abstract was deemed relevant, the full text was examined by 2 independent reviewers. Data were abstracted from the studies included at the full-text level. Abstraction elements included study characteristics such as time period and location, population characteristics such as age and health status, exposure including type of dietary pattern and/or dairy consumption, and reported outcomes of anxiety including method of diagnosis. After abstraction was completed by one reviewer, another reviewer independently checked all of the data elements for accuracy; conflicts were resolved by a senior reviewer.

### Study quality

Study quality of the included studies was appraised using the Academy of Nutrition and Dietetics Quality Criteria Checklist [10], which was specifically designed for nutrition studies. This checklist collects yes, no, not available (NA), or unclear responses to 10 questions assessing various domains in which bias can arise in a study (e.g., inclusion and exclusion criteria, data collection, data analysis, and conflicts of interest). The tool was modified for this SLR as follows: first, the question regarding handling of withdrawals was deemed not relevant for cross-sectional and case-control studies; second, the question about blinding was only answered for RCTs. Based on responses to the 10 questions, a study was then given a final evaluation of positive, negative, or neutral; a study was given a positive rating if the majority of the answers were ‘yes’ including criteria 2,3,6, and 7; a neutral rating if the majority were yes or there were some NA/unclear but criteria 2,3,6, and 7 were not all ‘yes’; or a negative rating if majority of the answers were ‘no’.

## Results

### Article identification

**Fig 1:**
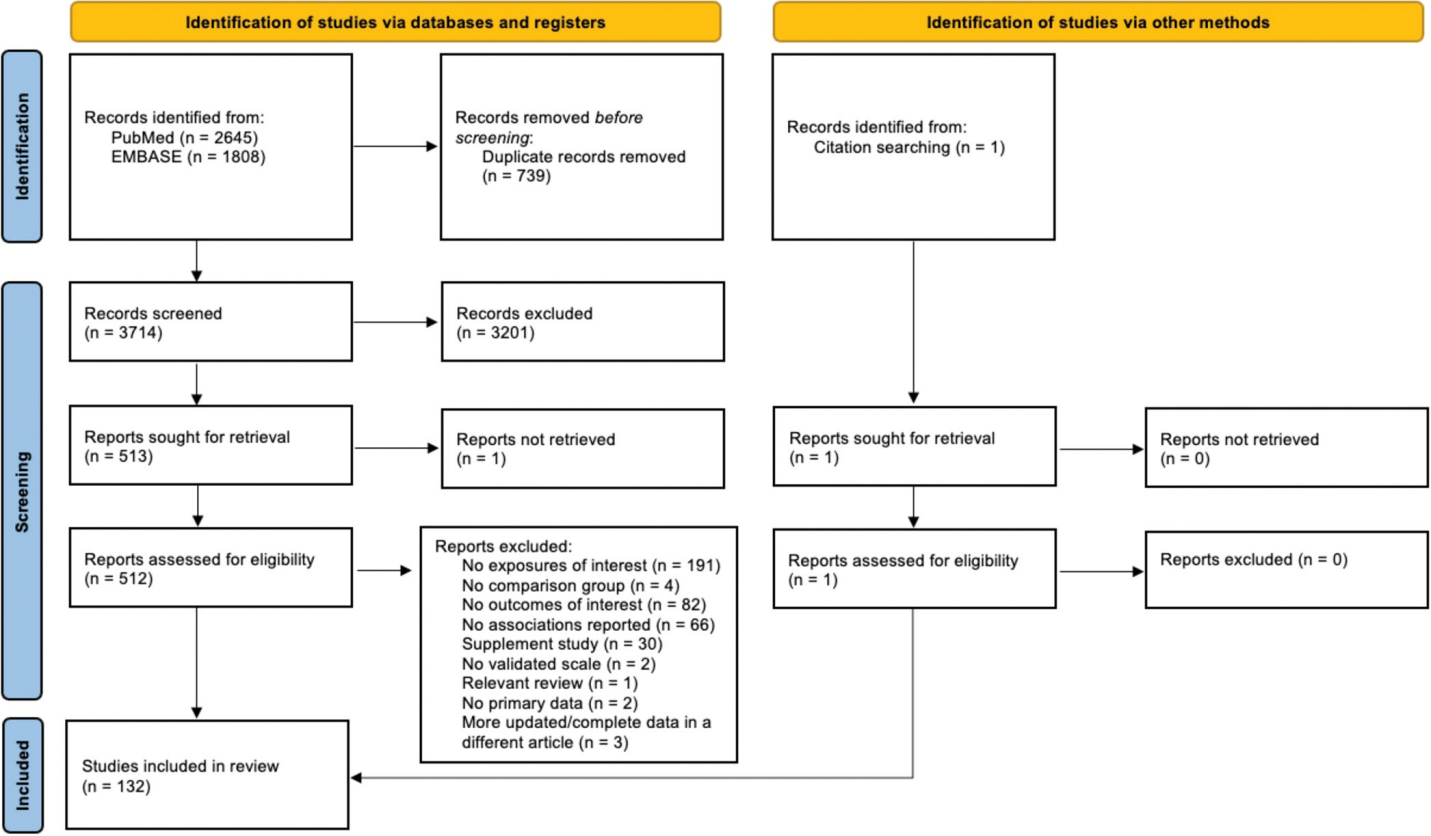
PRISMA study flow diagram.

The PRISMA flow diagram, detailing study inclusion and exclusion at each stage, is shown in Fig 1. See S1 File for the PRISMA checklist. Searches yielded 4,453 hits; after de-duplication across databases, 3,714 articles were screened at the title and abstract level. References cited in one relevant review were checked and one additional study was identified. There were 513 studies reviewed at the full-text stage. Of the 381 excluded at the full-text stage, 191 did not have any exposures of interest, 82 had no outcomes of interest, 66 did not report on the association between dietary pattern/dairy and anxiety, 30 only examined effects of supplements, 4 had no comparison group, 3 had overlapping data, 2 did not use a validated scale for anxiety, 2 had no primary data, and 1 was a relevant review. One hundred and thirty-two studies meeting the pre-defined PICOS criteria were thus included.

### Study quality of included studies

Among the 132 studies, 59 were given a positive quality rating, 72 a neutral rating, and one a negative rating. The responses for the questions in the checklist across the studies are summarized in S1 Fig. Overall, studies clearly defined the research question, selected the appropriate population, handled withdrawals, and defined exposures and outcomes well. Study quality issues were apparent in the appropriateness of the statistical analyses, specifically with confounder adjustment, and adequate reporting of methods and results.

### Study and population characteristics

The characteristics of the included studies are provided in S2 Table. Eighty-one (61%) of the 132 studies were cross-sectional, 22 (17%) were prospective cohort studies, 21 (16%) were clinical trials, 4 (3%) were case-control and 4 (3%) were other study designs. Study locations varied with the top three being Iran (n = 29), US (n = 18), and Australia (n = 16). Five studies were conducted across multiple countries. One hundred and eighteen studies included adult populations, while 7 were conducted among children and 7 included both adult and child populations. Seventy-three studies consisted of general populations, while 59 studies investigated specific populations with preexisting comorbidities such as diabetes or Celiac disease.

Many different dietary patterns were explored in these studies. Patterns examined in at least 3 studies included: Mediterranean (n = 21), gluten-free (n = 20), Healthy Eating Index (HEI) (n = 10), traditional (n = 10), diet quality score (n = 9), vegetarian and/or vegan (n = 9), Western (n = 8), low-carb (n = 7), FODMAP (fermentable oligosaccharides, disaccharides, monosaccharides and polyols) (n = 6), “healthy” (n = 6), omnivore (n = 6), plant-based diet (n = 4), and fish diet (n = 3). Dairy product consumption was examined in 19 studies.

Anxiety was measured using various validated tools with the most common being the Hospital Anxiety and Depression Scale (n = 33), Depression Anxiety Stress Scale (n = 28), State-Trait Anxiety Inventory (n = 22), Generalized Anxiety Disorder (n = 13), and Profile of Mood States (n = 8). Herein, the focus will be on the studies that evaluated dietary patterns examined in at least 3 studies.

### Dietary patterns and anxiety

**Fig 2:**
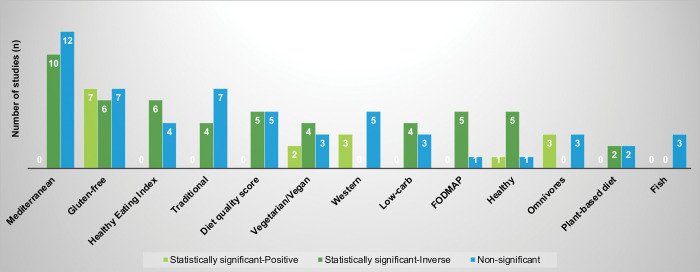
Associations observed among studies examining various dietary patterns and anxiety (N = 97 studies)^a^. ^a^ Of the 132 studies examining dietary patterns and anxiety, 97 studies included dietary patterns besides dairy that were examined in three or more studies and are included in this figure. Four studies were counted twice because there were relationships in different directions when subgroups were considered. Sixteen studies examined two or more dietary patterns, besides dairy.

Associations observed among studies examining various dietary patterns and risk of anxiety are shown in Fig 2 and S2 Table. Twenty-one studies examined the association between Mediterranean diet and anxiety. Of these, 9 showed statistically significant inverse associations [11–19], 11 were not statistically significant [20–30], and one study showed a significant inverse association for girls while no association was observed for boys [31]. Gluten-free diet and anxiety were evaluated in 20 studies (with many conducted among Celiac disease patients) and showed inconsistent results with 7 significant positive [32–38], 6 significant inverse [39–44], and 7 non-significant associations [45–51]. Of the 10 studies examining HEI and anxiety, 6 reported a significant inverse association [52–57] while 4 did not show any significant associations [26, 58–60]. Ten studies evaluated the association between a ‘traditional’ diet, which varied based on the geographic location, and anxiety, with 3 reporting a significant inverse association [61–63], 6 showing no association [64–69], and one study noting a significant inverse association for women while no association was noted for men [70]. The relationship between diet quality score and anxiety was examined in 9 studies, with 4 studies showing significant inverse associations [52, 71–73], 4 indicating no association [7, 63, 74, 75], and 1 study reporting a significant inverse association among women but no association among men [70]. Associations between a vegetarian/vegan diet and anxiety in 9 studies were inconsistent, with 2 reporting positive significant associations [62, 76], 4 noting a significant inverse association [77–80], and 3 indicating no significant associations [34, 47, 81]. Eight studies examined a Western diet and anxiety with 3 noting a significant positive association [65, 67, 82] and 5 not reporting an association [63, 64, 68, 70, 83].

The relationship between a low-carb diet and anxiety was explored in 7 studies, with 4 indicating a significant inverse association [84–87] and 3 not showing any association [88–90]. Of the 6 studies examining FODMAP diet and anxiety, 5 showed a significant inverse association [91–95], while the remaining study did not observe any significant associations [96]. Healthy diet and anxiety were evaluated in 6 studies, with 4 studies showing a significant inverse association [67, 97–99], one reporting no association [64], and one study noting a significant positive association for men and a significant inverse association for women [63]. Associations between an omnivore diet and anxiety were evaluated in 6 studies, with 3 reporting a significant positive association [69, 77, 78] and 3 showing no association [81, 100, 101]. Plant-based diet and anxiety were examined in 4 studies, with 2 indicating a significant inverse association [102, 103] and 2 reporting no significant associations [100, 104]. Lastly, the relationship between a diet high in fish consumption and anxiety was explored in 3 studies with all 3 reporting non-significant associations [83, 105, 106].

**Fig 3:**
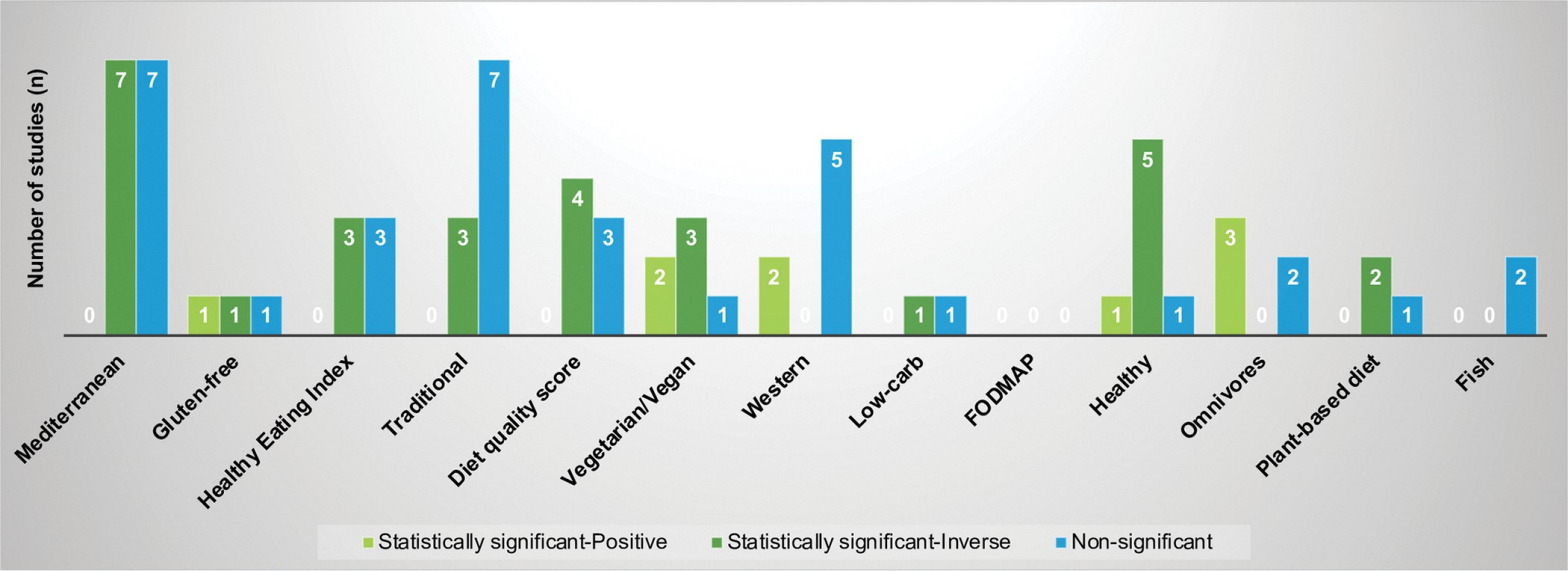
Associations observed among studies examining various dietary patterns and anxiety in general populations (N = 50 studies)^a^. ^a^ Of the 73 studies conducted among general populations, 50 studies included dietary patterns besides dairy that were examined in three or more studies and are included in this figure. Four studies were counted twice because there were relationships in different directions when subgroups were considered. Twelve studies examined two or more dietary patterns, besides dairy.

Of the 73 studies conducted in the general population (i.e., not selected for any specific health condition), 50 studies included dietary patterns besides dairy that were examined in three or more studies (Fig 3, S2 Table). The associations observed among these general population studies were similar to the patterns described above when all populations were considered.

**Fig 4:**
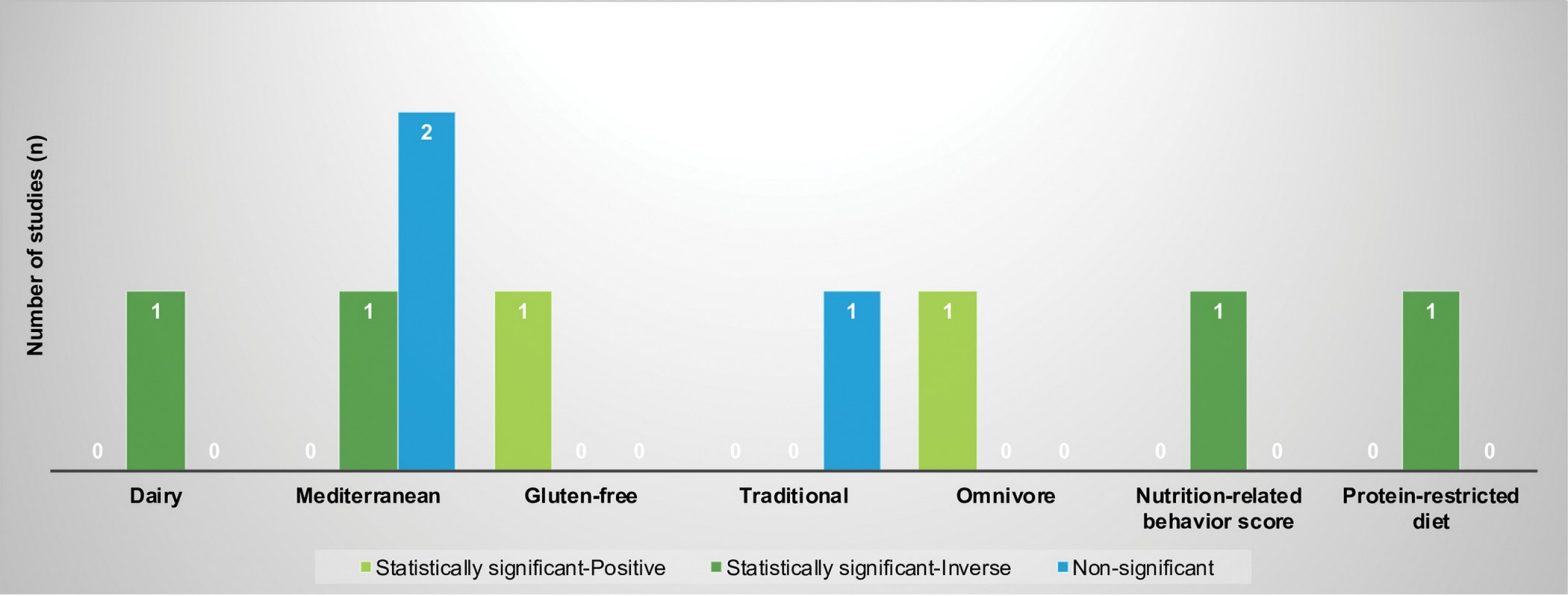
Associations observed among studies examining various dietary patterns and anxiety in children (N = 7 studies)^a^. ^a^ One study was counted twice because there were relationships in different directions when subgroups were considered. One study examined two dietary patterns.

Among the 7 studies that included only children, associations between dairy (n = 1), Mediterranean (n = 2), gluten-free (n = 1), traditional (n = 1), omnivore (n = 1), nutrition-related behavior score (n = 1), and protein-restricted diet (n = 1) and anxiety were examined, as shown in Fig 4 and S2 Table. Of the 2 studies examining the Mediterranean diet, one did not show any association [27] while one study reported a significant inverse association for girls and no association for boys [31]. Significant positive associations were reported for a gluten-free diet [35] and an omnivore diet [69]. Significant inverse associations were observed for dairy consumption [107], nutrition-related behavior score [108], and a protein-restricted diet [109]. There was no association between a traditional diet and risk of anxiety [69].

### Dairy consumption and anxiety

**Fig 5:**
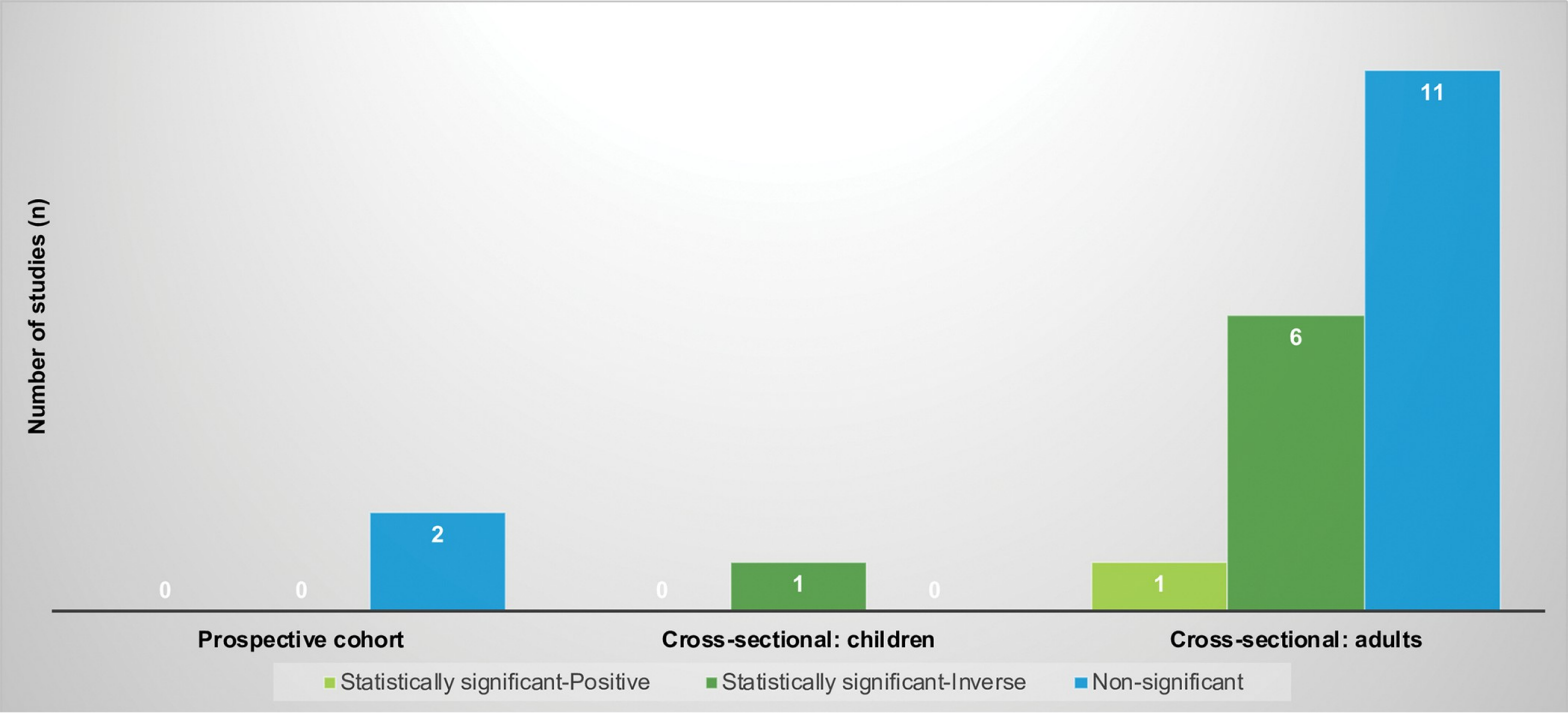
Associations observed among studies examining dairy consumption and anxiety (N = 19 studies)^a^. ^a^ Two studies were counted twice because there were relationships in different directions when subgroups were considered.

The relationship between dairy consumption and anxiety was examined in 19 studies, as shown in Fig 5 and Table 1. One study was a prospective cohort study conducted among adults and did not report any significant associations between dairy intake and the risk of anxiety [59]. The remaining 18 studies were cross-sectional, including one study conducted among children which reported a statistically significant inverse association between dairy consumption and anxiety; i.e., more dairy intake was associated with lower risk of anxiety [107]. Among the 17 cross-sectional studies of adult populations, 4 reported a statistically significant inverse association [5, 13, 110, 111], 11 showed no association [6, 7, 14, 16, 57, 60, 106, 112–115], and 2 noted mixed associations [12, 104]. One study noted a statistically significant inverse association for women, but no association for men [12], while another study indicated a statistically significant inverse association for low-fat dairy intake and a positive association for high-fat dairy consumption [104].

**Table 1:** Characteristics and effect measures of studies examining dairy consumption and anxiety (N = 19 studies).

Author (Year)	Study Country	Study Design	Study Period	Study Size (N)	Population Age, Type^a^	Anxiety Scale used for Assessment	Dairy Product Exposure	Reported effect measures with 95% CI for risk of anxiety		Study Quality
Du (2022) [112]	United States	Cross-sectional	Apr-May 2020	1233	Adults, General	GAD-7	Dairy	β coefficient for anxiety score: -0.013; p = 0.662		Positive
Lee (2022) [6]	Canada	Cross-sectional	Aug 2018- Feb 2019	141	Adults, General	DASS	Dairy and alternatives	β coefficient (95% CI) for anxiety score: -1.83 (-3.70, 0.03)		Neutral
Alkhatatbeh (2021) [113]	Jordan	Cross-sectional	Sept 2019-Jan 2020	1000	Adults, General	HADS	Dairy calcium intake	β coefficient for anxiety score: 0.03; p = 0.20		Neutral
Boaz (2021) [12]	Multi-country	Cross-sectional	NR	3271	Adults, General	GAD-7	Unsweetened dairy	Association between servings/day and anxiety score (rho, p): Women: −0.07, 0.001; Men: 0.01, 0.728; p = 0.04		Neutral
Ghodsi (2021) [114]	Iran	Cross-sectional	Oct-Dec 2019	230	Adults, General	DASS	Total dairy	OR (95% CI) for risk of anxiety by tertiles of consumption: T1: 1.00 T2: 0.92 (0.38, 2.24); p = 0.864 T3: 0.60 (0.22, 1.63); p = 0.323		Neutral
							Low dairy	OR (95% CI) for risk of anxiety by tertiles of consumption: T1: 1.00 T2: 0.93 (0.38, 2.24); p = 0.873 T3: 0.42 (0.14, 1.26); p = 0.123		
Gomes (2021) [59]	Brazil	Prospective cohort	NR	3331	Adults, General	Generalized anxiety disorder-version not provided	Milk and dairy products	OR (95% CI) for risk of anxiety: 0.95 (0.82, 1.10)		Neutral
Mahdavifar (2021) [5]	Iran	Cross-sectional	Sept 2014-Mar 2016	7387	Adults, General	DASS	Low-fat dairy product	OR (95% CI) T1: 1.00 T2: 0.89 (0.73, 1.08) T3: 0.79 (0.64, 0.96) p-trend = 0.02		Positive
							High-fat dairy product	OR (95% CI) for anxiety by tertile of consumption: T1: 1.00 T2: 0.70 (0.57, 0.86) T3: 0.82 (0.66, 1.02) p-trend = 0.04		
							Total dairy	OR (95% CI) for anxiety by tertile of consumption: T1: 1.00 T2: 0.83 (0.68, 1.01) T3: 0.73 (0.59, 0.91) p-trend = 0.005		
							Low-fat milk	OR (95% CI) for anxiety by tertile of consumption: T1: 1.00 T2: 0.80 (0.62, 1.03) T3: 0.82 (0.68, 0.99) p-trend = 0.03		
							High-fat milk	OR (95% CI) for anxiety by tertile of consumption: T1: 1.00 T2: 0.93 (0.70, 1.23) T3: 0.82 (0.68, 1.00) p-trend = 0.05		
							Total Milk	OR (95% CI) for anxiety by tertile of consumption: T1: 1.00 T2: 0.75 (0.61, 0.92) T3: 0.72 (0.58, 0.88) p-trend = 0.002		
Marck (2021) [7]	Australia	Cross-sectional	2016–2017	1490	Adults, Specific: Multiple sclerosis	HADS, Severity of MS symptoms (MSSysMS)	Overall dairy (yes vs. no)	β coefficient for anxiety (95% CI): 0.2 (-0.6, 1.1)		Neutral
Sadeghi (2021) [13]	Iran	Cross-sectional	2010	3172	Adults, General	HADS	Dairy	OR (95% CI) for anxiety by quartile of consumption: Q1: 1.00 Q2: 0.70 (0.50, 0.98) Q3: 1.15 (0.84, 1.58) Q4: 0.71 (0.50, 1.00) p-trend = 0.38		Positive
Vassou (2021) [14]	Greece	Cross-sectional	2002–2012	853	Adults, General	STAI	Dairy	Servings/week (mean ± SD): High irrational beliefs and low-STAI: 11.6 ± 4.4 High irrational beliefs and high-STAI: 12 ± 5.3 p = 0.81		Neutral
Alkhazrajy (2020) [110]	Iraq	Cross-sectional	Jan-Jul 2018	400	Adults, General	HADS	Dairy products	OR (95% CI) for risk of anxiety: 0.74 (0.55, 0.92); p = 0.041		Neutral
Daneshzad (2020) [104]	Iran	Cross-sectional	NR	230	Adults, Specific: Type 2 diabetes	DASS	Low fat dairy products	Mean (SE) grams/day: Anxiety = No: 195.2 (11.48) Anxiety = Yes: 118.3 (16.19) p<0.0001		Positive
							High fat dairy products	Mean (SE) grams/day: Anxiety = No: 90.96 (10.25) Anxiety = Yes: 168.3 (14.46) p<0.0001		
Gibson-Smith (2020) [16]	Netherlands	Cross-sectional	NR	1634	Adults, Specific: Lifetime depressive or anxiety disorder	Composite International Diagnostic Interview, Beck Anxiety Inventory	High fat dairy after correcting for all other food groups	β coefficient for anxiety (95% CI): 0.00 (-0.05, 0.05), p = 0.99		Positive
Khanna (2020) [107]	India	Cross-sectional	NR	546	Children, General	Child Behavior Checklist	Consumption of milk as usual breakfast on school days, yes vs. no	Mean (SE) anxiety score by milk consumption:		Neutral
								Males: Milk = No: 60.71 (0.74) Milk = Yes: 57.17 (0.58) p = 0.0002	Females: Milk = No: 62.01 (0.71) Milk = Yes: 58.88 (0.75) p = 0.0028	
								Overall: p<0.0001		
Sangsefidi (2020) [111]	Iran	Cross-sectional	NR; YaHS Recruitment phase was Sept 2014-Mar 2016	9965	Adults, General	DASS	Milk	OR (95% CI) for risk of anxiety: None: 1.00 1–2 glasses/week: 0.73 (0.61, 0.87) >2 glasses/week: 0.98 (0.80, 1.20)		Neutral
							Yogurt	OR (95% CI) for risk of anxiety: None: 1.00 1–2 glasses/week: 0.72 (0.52, 0.98) >2 glasses/week: 0.54 (0.40, 0.73)		
Taylor (2020) [60]	United States	Cross-sectional	NR	133	Adults, General	DASS	HEI Dairy Score	β coefficient for anxiety ± SE (95% CI) 0.01 ± 0.03 (-0.05, 0.07), p = 0.72		Positive
Rintamäki (2014) [115]	Finland	Cross-sectional	2000–2001	5504	Adults, General	Composite International Diagnostic Interview	Dairy products total	Mean grams/day (95% CI) Anxiety = No: 579 (566–592) Anxiety = Yes: 536 (425–648) p = not significant		Neutral
							Milk	Mean grams/day (95% CI) Anxiety = No: 323 (314–334) Anxiety = Yes: 352 (256–447) p = not significant		
							Fermented milk products	Mean grams/day (95% CI) Anxiety = No: 186 (179–193) Anxiety = Yes: 117 (67–166) p = not significant		
							Cheese	Mean grams/day (95% CI) Anxiety = No: 44 (43–46) Anxiety = Yes: 42 (33–51) p = not significant		
Forsyth (2012) [57]	Australia	Cross-sectional	2006–2008	109	Adults, Specific: Depression/ anxiety	DASS	Milk	Pearson’s correlation coefficient with DASS score: -0.03; p>0.05		Positive
Yannakoulia (2008) [106]	Greece	Cross-sectional	2003	853	Adults, General	STAI	Dairy	Difference in servings/week by tertile of STAI score: Women: p = 0.536 Men: p = 0.654		Positive

^a^ Studies were defined as a specific population if the study was selected based on existing disease such as diabetes.

DASS, Depression, Anxiety, and Stress Scale; GAD-7, Generalized Anxiety Disorder 7; HADS, Hospital Anxiety and Depression Scale; MS, multiple sclerosis; OR, odds ratio; STAI, State-Trait Anxiety Inventory.

Of the 19 studies on dairy consumption, 5 studies evaluated milk and anxiety [5, 57, 107, 111, 115]. In one Indian study, consumption of milk as breakfast on school days was associated with lower anxiety for both males (p = 0.0002) and females (p = 0.0028) [107]. There was a significant inverse association observed between consumption of 1–2 glasses/week (OR = 0.73; 95% CI: 0.61, 0.87) and anxiety in one Iranian study, but not among those who consumed >2 glasses/week (OR = 0.98; 95% CI: 0.80, 1.20) [111]. Another Iranian study noted significant inverse trends across tertiles of total milk consumption (p-trend = 0.002) and low-fat milk consumption (p-trend = 0.03) and anxiety while the trend with high-fat milk was of borderline statistical significance (p-trend = 0.05) [5]. The remaining 2 studies showed non-significant association [57, 115].

One Finnish study examined cheese and anxiety and did not report a significant association [115]. There was a significant inverse association observed between yogurt consumption and risk of anxiety in one Iranian study (None: reference, 1–2 glasses/week: OR = 0.72 [95% CI: 0.52, 0.98), >2 glasses/week: OR = 0.54 [95% CI: 0.40, 0.73]) [111]. Fermented milk products were evaluated in one Finnish study and no associations were reported [115]. Three studies examined high-fat dairy products, with one Iranian study reporting a significant inverse association for those in tertile 2 (OR = 0.70; 95% CI: 0.57, 0.86) but not in tertile 3 and a significant trend (p-trend = 0.04) [5]. Another Iranian study reported a significant positive association with anxiety (p<0.0001) [104], while there was no association for high-fat dairy and anxiety in one Netherlands study after correcting for other food groups (p = 0.99) [16]. The association between low-fat dairy products and anxiety was evaluated in 3 Iranian studies with mixed results. One study did not observe any significant associations [114] and two studies observed significant inverse associations [5, 104]. None of these studies specified the precise levels of dairy consumption.

## Discussion

This SLR identified and assessed 132 studies which examined the association between dairy consumption and/or various dietary patterns and the risk of anxiety with no restrictions on populations or geographic location. Of 19 studies evaluating dairy consumption, 7 reported a significantly lower risk of anxiety associated with increased dairy intake, while significant associations were not observed in 12 studies. Evidence for the other dietary patterns including but not limited to HEI, Mediterranean, and gluten-free diets were mixed but generally indicated a reduced risk of anxiety with greater adherence to a specific diet (e.g., more adherent to the Mediterranean diet), similar to that of dairy consumption. Inconsistencies in the direction of associations could be due to differences in study populations, time periods, locations, anxiety scales, dietary assessment, and confounder adjustment. For example, many of the study populations were specific to individuals with chronic conditions that may affect both dietary habits and risk of anxiety, such as type 2 diabetes, irritable bowel syndrome, Celiac disease, hypertension, and polycystic ovarian syndrome. Moreover, the statistical analyses differed between studies, complicating interpretation of the results across studies (e.g., odds ratios for risk of anxiety by tertile of dairy consumption, p-value for difference in anxiety score by binary measure of dairy consumption, p-value for difference in dairy servings/week among those with and without anxiety).

Most of the studies identified in this SLR were cross-sectional meaning that exposure and outcome were measured at the same point in time, making it difficult to demonstrate a temporal cause-and-effect relationship between dairy and/or dietary patterns and anxiety. As diet is a complex exposure, which involves many components that simultaneously affect health and given that individual habits and food composition change over time and that people may eat differently if they are in an anxious state, reliable measurement of dietary intakes across individuals and various populations is critical [116]. Furthermore, diet collection instruments have the potential for recall bias resulting in omission of foods consumed by individuals and/or errors in nutrient compositions from food composition tables [116]. Many of the studies in this SLR used food frequency questionnaires or other similar methods to ascertain dietary exposures and did not estimate serving sizes, so precise comparisons could not be made. Larger prospective cohort studies are needed to supplement these findings, as prospective cohorts can collect dietary intakes repeatedly over time and, as a result, minimize recall and selection biases; thus ultimately, providing stronger evidence for inferences around causality.

While the study quality metrics reflected that some studies did not adjust for confounders in their analyses, among studies with adjustment, the common confounders were age, sex, socioeconomic status, BMI, physical activity and education. Very few studies controlled for energy intake. Variables such as use of medications prescribed for anxiety, previously diagnosed anxiety, caffeine usage, and consumption of other dietary supplements that could impact diet quality were not routinely captured or accounted for in the studies. Further, social and cultural factors related to diet may have varied within and across the populations studied. Failure to adjust for these variables may confound any relationship as people who eat healthier may tend to have better mental and physical health. Lack of confounding adjustment could be masking true associations between dairy and/or dietary patterns and anxiety. Prospective studies with robust confounder adjustment both at the design and analysis stages would strengthen the existing literature surrounding diet and anxiety.

Though the majority of the 19 studies reporting on dairy consumption and anxiety reported non-significant associations, the 7 studies that did report significance were in the same inverse direction (i.e., higher dairy intake was associated with lower risk of anxiety). While this evidence cannot support a causal conclusion given the lack of temporality in the cross-sectional nature of the studies, these findings present interesting hypotheses that merit further evaluation in prospective observational studies or clinical trials. Further, there may be variability in the components of the exposure that could be impacting the study outcome (whole fat versus low-fat, fermented products, etc.). Many essential nutrients such as vitamin D, zinc, protein, riboflavin, and vitamin B12 are found in milk and other dairy products [117]. A recently published umbrella review identified 43 meta-analyses evaluating 95 outcomes associated with zinc intake and reported that higher dietary zinc intake results in an overall lowered risk of depression [118]. This inverse association was also observed in a recent systematic review of vitamin D deficiency and psychophysiological variables which reported a reduction in depression and anxiety symptoms associated with an increase in vitamin Ds [119]. Thus, the inverse association between dairy and anxiety reported in some studies is concordant with the existing literature describing the links between specific dairy components and improved health risks. Further research is needed to fully elucidate the specific effects of dairy products on mental health outcomes.

Several mechanisms have been proposed regarding the pathways in which diet may influence mental health. A recent scoping review of diet and anxiety indicated a habitual diet impacts the gut microbiome which in turn affects the modulation of the gut peptide production involved in the gut-brain axis and neurotransmitter synthesis [2]. Additionally, a review of diet and depression acknowledged the majority of evidence in nutritional psychiatry is from preclinical animal studies and noted that diet impacts mental health through modulation of pathways such as oxidative stress, gut microbiota, epigenetics, neurogenesis, inflammation, and the hypothalamic-pituitary-adrenal axis [120]. One proposed mechanism for the impact of dairy on anxiety is through serotonin production, which requires tryptophan and calcium, available in dairy products [112]. Since low levels of serotonin have been linked with higher anxiety, increasing serotonin production through dairy consumption could potentially improve mental health [112]. Interventional studies with clinically diagnosed human populations are needed to better elucidate the complex pathways associating diet with health outcomes such as anxiety.

There are several limitations to this systematic review. The use of food frequency questionnaires in many of the studies might not be accurately reflecting individuals’ dietary patterns given the potential for measurement error associated with participant recall. There was also a lack of consistency in the specific anxiety instruments used across studies. Individuals with preexisting anxiety were not excluded, which could create bias in the effect measures observed in the studies. Due to the design of the studies obtained in the course of this SLR, we were unable to parse out whether any observed associations were between a specific dietary pattern or rather the commitment to following an eating pattern in general. Finally, most studies in this landscape are cross-sectional in nature which both precludes the collection of dietary intakes over time and limits the ability to demonstrate a cause-and-effect relationship between dairy or various dietary patterns and anxiety. Strengths of this SLR include the use of rigorous methodology including the PRISMA guidelines, assessment of study quality, 100% quality control of the abstraction and study quality determinations, and examination of multiple dietary patterns across the globe with no restrictions on populations.

This SLR suggests the potential for dairy and various dietary patterns to impact the risk of anxiety across a variety of populations. Future studies with longitudinal design, clearly defined eligibility criteria, and strong dietary assessment methodology are needed to better understand the complex relationships between diet and anxiety. The benefits of such knowledge to public health are vast as diet is a modifiable risk factor and tailored interventions have the potential to improve the mental well-being of millions of individuals.

## Supporting information

S1 File PRISMA checklist 2020. (DOCX)

S1 Fig Summary of study quality scores for the included studies (N = 132). (TIFF)

S1 Table Literature search strategy. (XLSX)

S2 Table Characteristics and effect measures of the included studies, organized by publication year (N = 132 studies). (XLSX)
