# Supplementary figures and images for: Dietary eating patterns, dairy consumption, and anxiety: A systematic literature review
Source: PLoS One. 2023 Dec 28;18(12):e0295975. doi: 10.1371/journal.pone.0295975 (PMC10754443; doi:10.1371/journal.pone.0295975)

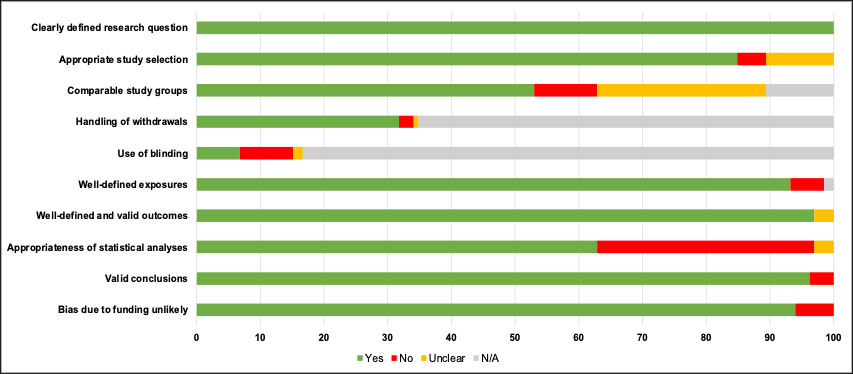

Supplement: S1 Fig — (TIFF) [file pone.0295975.s002.tiff]
